# Medical and Physical Researches; or, Original Memoirs in Medicine, Surgery, Physiology, Geology, Zoology, and Comparative Anatomy. Illustrated with Plates, Containing 160 Figures

**Published:** 1836-10

**Authors:** 


Art. X.—*Medical and Physical Researches; or, Original Memoirs in Medicine, Surgery, Physiology, Geology, Zoology, and Comparative Anatomy. Illustrated with Plates, containing* 160 *Figures.* By R. Harlan, m.d. f.l.s. London; Surgeon to the Philadelphia Almshouse Hospital; Professor of Comparative Anatomy, &c.—Royal 8vo. pp. 653. *Philadelphia,* 1835.

This is one of the best specimens of the bookcraft, so far as regards the mechanical execution, that we have ever seen from the American press. Dr. Harlan is well and deservedly known for his attention to natural science more especially: his contributions to this department of knowledge, as well as to that of medicine, are spread through various periodical publications, and it seems chiefly for the purpose of embodying them in one volume that the work before us has been composed. The reader, consequently, has not to look for much novelty in its pages.

The chief medical papers are the following:

1. "An Inquiry into the Functions of the Brain of Man, and in the lower order of Animals; delivered as a Lecture before the Academy of Natural Sciences of Philadelphia in 1824;" in which the author concludes "that nothing is more clearly demonstrated than that the brain, the material organ of the understanding, is as complex in its composition, and made up of as many distinct and separate organs as there are special faculties of the mind;" and that "man is far exalted above the brutes, by a superior degree of perfection in his intellectual faculties, by a greater power of restraint over his instincts, and by readier methods of combination and of communicating his ideas and feelings, rather than by a difference in the nature of his mental constitution." (P. 439.)

2. "Experiments to ascertain the Operation of various Poisons on living Vegetables, performed in 1830 and 1831." These were undertaken with the view of confirming or disproving the experiments and deductions of Mr. Princep and others regarding the poisonous effects exerted on certain plants, or on animals, by different agents. The experiments were performed by Dr. Harlan, in the presence of Dr. Moore and Mr. Featherstonehaugh. The plants to which the poisons were applied were, Palma Christi, Datura stramonium, Nicotiana tabacum, Balsamina impatiens, Brassica, Geranium, and Carduus benedictus. The poisons used were, Oleum tabaci, Oxidum arsenici, Extractum stramonii, Extractum cicutæ, corrosive sublimate in solution, oil of turpentine, and a strong solution of opium. Each of these poisons was introduced separately into the circulation of individual plants, by incisions made in the stems under the leaves, and by separate applications of them to the roots, by infusion, and by powder also in the case of arsenic. In some instances the poisons were placed around the roots only: this was done with the corrosive sublimate, arsenic, spirit of turpentine, and oil of tobacco. In no instances was any one of the plants poisoned: one of the young geraniums faded, after constantly impregnating the earth about its roots for three days; but this was ascribed to the soil being rendered unfit for the support of vegetable life.

3. In a paper entitled "Experiments performed on living Animals, to prove that the Circulation of the Blood through the Lungs is immediately and entirely suppressed during Expiration," Dr. Harlan maintains the ancient doctrine, that there is a mechanical impediment to the circulation from the right to the left side of the heart, owing to the extremities of the arteries and veins being subjected to compression; an opinion for which he will find but few supporters. He farther affirms that, in every case of asphyxia, there is a negation of oxygen, and consequent paralysis of the lungs. Such paralysis is therefore, he conceives, in every instance, the *immediate* cause of death.

"For no sooner does the paralysis of the lungs take place, than that power by which the life of animals is produced and continued, and which I suppose to be derived from the atmosphere, is put a stop to, and the blood, which animates all the solids, no longer receiving its supply, death, or the total extinction of the vital principle, is the consequence." (P. 455.)

4. "Report of the Committee of the Academy of Medicine of Philadelphia, on the Means by which Absorption is effected." This is the well-known Report of Messrs. Harlan, Lawrence, and Coates, on the question, to be investigated by experiment, "whether the veins absorb, and whether positive evidence can be obtained that the lymphatics perform that function, or that the lacteals absorb any thing but chyle." The results are referred to in most of the works on Physiology.

5. It has been a disputed point whether the human intestinal worms ever perforate the intestines, whilst it is admitted that the Strongylus armatus, an intestinal parasite of the horse, occasionally penetrates the canal, and gets into the arteries, so as to produce aneurism. Such a case is given by Dr. Harlan. A colt, one year old, died suddenly. On dissection, the following appearances presented themselves:

"We first examined the brain, which we found healthy, though there was serous effusion within the sheath of the medulla spinalis. On opening the abdomen, we were surprised by the effusion of at least two bucketsful of fluid black blood. This excited my attention, as I had before met with a similar case, where the cause of the mischief had not been investigated. Continuing the dissection, we came to an immense tumour lying over the right kidney, occupying the whole of the right lumbar and iliac regions, and which was filled with grumous coagulated blood. On removing this, we found the sac confusedly connected to the mesentery, the aorta, and surrounding parts. We next dissected away the aorta abdominalis, above and below the sac, and, on opening the artery, we observed the internal coat near that portion which gives off the mesenteric artery much diseased, and considerably enlarged; the superior mesenteric artery was particularly enlarged; the internal coat both of the mesenteric and of the aorta, for several inches, being nearly destroyed, and of a black colour. On examining more accurately the portion of the aorta below the tumour, we discovered the cause of the whole mischief,—viz. *a great number of small worms,* from a quarter to three-quarters of an inch in length, attached to the internal coat of this portion of the artery, giving it truly a *worm-eaten* appearance. The worms, on examination by Mr. Thomas Lay, proved to be an intestinal species, common in the horse: they must therefore have eaten their way from the small intestines of the animal into the mesenteric artery, from whence they continued their course to the aorta, destroying, as they proceeded, the internal coat. As their presence in this unnatural situation produced inflammation, coagulated lymph was thrown out between them and the current of blood, by which they became in a manner *incisted* (encysted). The artery became enlarged by their irritation, and formed a species of aneurism, particularly of the mesenteric artery, which eventually burst suddenly, after the animal had eaten a full meal, and produced immediate death, the usual termination of such cases." (P. 554.)

In addition to these, there are several papers of less interest, amongst which "Observations on the Malignant Cholera, as it occurred in

Philadelphia, in 1832;" and an experiment showing that sperm injected into the cellular membrane at the upper part of the thigh of a bitch, in heat, did not fecundate, are perhaps amongst the most important.

Prefixed to the work is an introduction, containing some judicious observations "on the Affiliation of the Natural Sciences," and more especially on their relation to medicine. It is to be regretted, however, that here, as elsewhere, the author should not have bestowed the necessary pains to prevent typographical and other inaccuracies, which are a blemish to the work, creditable as it is in all other respects. Thus, at page xv., we find "the functions must be observed and compared in all the links in the great chain of beings to *whom* any modification of vitality has been imparted;" and, at page 597, we have, twice repeated the term *rupture uteri.* Several of the names, too, are so modified as to be scarcely recognizable; as, for example, Fife (Fyfe), Shneider (Schneider), Tidyman and Tydiman (Tiedemann), Malacaine (Malacarne), Pausanius (Pausanias), Atheneus (Athenæus), Lyelle and Lyle (Lyell). Most, if not all, of these inaccuracies may be typographical, but they are not the less inaccuracies; and their number shows that due attention has not been bestowed by the author, which is the more surprising as the whole appearance and character of the work show that *effect* has been not a little studied.

